# Enhanced Plasmonic Biosensors of Hybrid Gold Nanoparticle-Graphene Oxide-Based Label-Free Immunoassay

**DOI:** 10.1186/s11671-018-2565-7

**Published:** 2018-05-16

**Authors:** Nan-Fu Chiu, Chi-Chu Chen, Cheng-Du Yang, Yu-Sheng Kao, Wei-Ren Wu

**Affiliations:** Laboratory of Nano-photonics and Biosensors, Institute of Electro-Optical Science and Technology, National Taiwan Normal University, No. 88, Sec. 4, Ting-Chou Road, Taipei, 11677 Taiwan

**Keywords:** Gold nanoparticle (AuNP), Graphene oxide sheet (GO), Localized surface plasmon resonance (LSPR), Immunoassays

## Abstract

In this study, we propose a modified gold nanoparticle-graphene oxide sheet (AuNP-GO) nanocomposite to detect two different interactions between proteins and hybrid nanocomposites for use in biomedical applications. GO sheets have high bioaffinity, which facilitates the attachment of biomolecules to carboxyl groups and has led to its use in the development of sensing mechanisms. When GO sheets are decorated with AuNPs, they introduce localized surface plasmon resonance (LSPR) in the resonance energy transfer of spectral changes. Our results suggest a promising future for AuNP-GO-based label-free immunoassays to detect disease biomarkers and rapidly diagnose infectious diseases. The results showed the detection of antiBSA in 10 ng/ml of hCG non-specific interfering protein with dynamic responses ranging from 1.45 nM to 145 fM, and a LOD of 145 fM. Considering the wide range of potential applications of GO sheets as a host material for a variety of nanoparticles, the approach developed here may be beneficial for the future integration of nanoparticles with GO nanosheets for blood sensing. The excellent anti-interference characteristics allow for the use of the biosensor in clinical analysis and point-of-care testing (POCT) diagnostics of rapid immunoassay products, and it may also be a potential tool for the measurement of biomarkers in human serum.

**Electronic supplementary material:**

The online version of this article (10.1186/s11671-018-2565-7) contains supplementary material, which is available to authorized users.

## Background

Carbon molecule-based materials such as carbon nanotubes [1, 2], carbon balls (buckminsterfullerene, C60) [3], two-dimensional graphene [4–6], and graphene oxide (GO) [7–11] have been widely used in biosensors. Among them, the two-dimensional sheet structure of graphene is an ideal material to allow for thin films with high conductivity [12, 13] and excellent optical permeability [14] characteristics and high biocompatibility [15, 16]. For these reasons, graphene-based material is widely use in biomedical and electrochemical sensing technology [17, 18]. In addition, the photoelectric type of biological sensing technology is mainly based on GO [19–21]. Because oxide groups can be adjusted to absorb and radiate a light band gap [22, 23], it is commonly used in fluorescence [24], surface plasmon resonance (SPR) [8–11, 19–21], and localized surface plasmon resonance (LSPR) [25, 26] sensing technology. In particular, GO has unique chemical functional groups (epoxy bridges, hydroxyl groups, pairwise carboxyl groups (carboxyl and carbonyl)) which improve the affinity and covalent bonding of biomolecules.

The synthesis of graphene material combined with nanoparticles (such as Pt, Au, Ag, Pd, and ZnO) has been widely studied for the development of new nanocomposite technology. In particular, the use of gold nanoparticles (AuNPs) has been used as a mechanism for the energy transfer of colorimetric and absorption spectroscopy. In addition, during the last decade, research on AuNPs in visible light has highlighted the unique plasmonic resonance characteristics. Because adjusting the size and shape of AuNPs can change the optical absorption wavelength shift, AuNPs can be used for enhanced plasma absorption and signal amplification [27, 28]. Therefore, AuNPs have been extensively used in a wide range of applications such as optoelectronic components because of their special optical and optoelectronic properties to enhance light extraction [29, 30] and light absorption reactions [31–33]. In addition, AuNPs are biocompatible, and they have been studied for their use in chemical sensing, biomedical imaging, cancer therapy [34, 35], drug carrier [32, 33], photo-thermal therapy [36–38], contrast agent [39], radiosensitizer [40], and biosensing [33, 41–43] applications.

The functionality of the AuNPs was modified by adding the cross linker to avoid oxidation and act as a carrier of phytochemicals or vectors; thus, this combination can increase biocompatibility and bioactivity [44–46]. Cross linkers such as cystamine (Cys) or 8-mercaptooctanoic acid (MOA) are activated by carboxylic acid-terminated thiol self-assembled monolayers (SAMs) on a modified Au surface. MOA binds to the Au surface through the thiol linker (-SH end) resulting in monolayers.

In addition, research on plasmon metal material has also been widely reported. For example, plasmonic metal core shell nanoparticles [47], nanostars [48], and fluorine-doped tin oxide nanoparticles [49] have been shown to enhance the energy band gap, which makes them desirable in solar cell and sensing applications.

Moreover, the use of AuNP-GO hybrids based on chemical synthesis [50–52] and electrostatic self-assembly [53] has been reported in sensor, energy, and catalytic applications. In recent years, an increasing amount of research has focused on the use of AuNP-GO hybrids in biosensors. These hybrids have been shown to be useful in the development of electrochemical [54–59] and surface-enhanced Raman scattering (SERS) [56, 59] platform technology to improve the application in biological assays. However, there are currently no relevant reports on the use of naked eye or colorimetric rapid immunoassay biosensor technology. For example, electrochemical DNA biosensors based on AuNP-GO hybrids have been used to detect breast cancer biomarkers to allow for an early diagnosis. With this biosensor, the detection limit (LOD) of 0.16 nM was obtained with a sensitivity of 378 nA/nM for the ERBB2 biomarker [54]. In addition, an AuNP dotted reduction graphene oxide (rGO-AuNP) nanocomposite-based electrochemical aptasensor has been used to selectively detect a concentration of 3,3′4,4′-polychlorinated biphenyls (PCB77) between 1 pg L^− 1^ and 10 μg L^− 1^, with a LOD of 0.1 pg L^− 1^ [55]. Moreover, AuNP-GO hybrids have been used as an electrochemical-based biosensor to detect hydrogen peroxide (H_2_O_2_), with food dynamic responses ranging from 0.1 to 2.3 mM, and an LOD of 0.01 mM [57]. Another good example is the utilization of AuNP-GO [56, 59] and AuNP-graphene [59] hybrids for SERS-based biosensors in diverse applications, as well as SERS-measured bioimaging.

In this study, we propose an alternative method of chemical synthesis and electrostatic self-assembly of AuNP-GO hybrids using layer-by-layer self-assembly. We also analyze the biological detection sensitivity of the modified combined AuNPs and GO sheets and their protein immune response. We designed two kinds of AuNP-GO-based protein label-free immunoassays and evaluated their response time and sensitivity in antigen-antibody interactions. The excellent sensing features of graphene-AuNP composites include ultra-high sensitivity and the affinity of biomolecule interactions that influence the detection of a diverse range of biomolecules with high specificity. These features imply that these composites have a promising role in future applications, and the potential to be the preferred route of disease detection in clinical diagnosis applications.

## Methods/Experimental

### Materials

Graphite was purchased from Graphene Supermarket (Graphene Laboratories Inc., Reading, MA, USA). GO sheets were obtained from graphite flakes by using a modified Hummer’s method [60] followed by ultrasonic shattering for 5 h for a flake size of 0.1–1 μm and thickness of 1.1 nm. Cystamine dihydrochloride (Cys, 96%), hydrogen tetrachloroaurate(III) trihydrate (HAuCl_4_·3H_2_O), ACS, 99.99% (metals basis), and Au 49.5% min were purchased from Alfa Aesar Co. (USA). Sodium citrate (HOC(COONa)(CH_2_COONa)_2_·2H_2_O) was purchased from J.T. Baker Chemical Co. (USA). Bovine serum albumin (BSA, SI-B4287, Sigma-Aldrich, USA), Anti-Bovine Albumin antibody produced in rabbit (antiBSA, SI-B1520, Sigma-Aldrich, USA), *N*-hydroxysuccinimide (NHS), and 1-ethyl-3-(3-dimethylaminopropyl)carbodiimide (EDC) were purchased from Sigma-Aldrich Inc. (USA). The immunoglobulins (Ig) of antiBSA antibody structure were produced by B lymphocytes and secreted into the plasma. The monomeric forms of Ig molecules were glycoproteins with a molecular weight of about 150 kDa. Each Ig monomer was capable of binding two antigen molecules. All reagents and solvents were used without further purification.

We used three different temperature conditions at 550, 400, and 100 °C with boiling times of 5, 5, and 120 minutes, respectively, to control reduction of the nanoparticles. These different temperatures reduced the nanoparticles to obtain the same absorption spectrum at 520 nm as clearly seen in Additional file 1: Figure S1.

### Synthesis of AuNPs

The method used to obtain AuNPs was based on the use of sodium citrate as a reducing agent to reduce tetrachloroaurate taurine ions in water. A volume of 15 mL of HAuCl_4_·3H_2_O solution containing 1 mM of Au was refluxed, and 1.8 mL of 38.8 mM sodium citrate (Na_3_C_6_H_5_O_7_) solution was added to the boiling (550 °C, 1100 rpm) solution. The reduction of the gold ions by the citrate ions was complete after 5 min, and the solution was further boiled for 30 min (400 °C, 900 rpm) and then left to cool to room temperature [36, 61, 62]. This method yields spherical AuNPs with an average diameter of about 15 nm, and a reduced concentration of 0.8 mL of 38.8 mM sodium citrate can be used to produce AuNPs with an average diameter of about 60 nm [63, 64]. The chemical reaction is as follows: HAuCl_4(aq)_ + C_6_H_5_O_7_Na_3(aq)_ → Au_(s)_ + CO_2_ + HCOOH.

### Preparation of GO Based on an Antigen Target

**Fig. 1:**
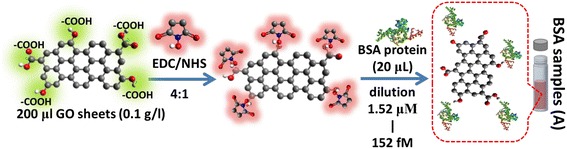
GO-BSA interaction. The carboxyl group of GO sheets can be activated using EDC/NHS reaction and preparation of the GO based on antigen target for GO-BSA

We designed an immunoassay method to prepare GO sheets as shown in Fig. 1. We used a 200-μL GO sheet solution at a concentration of 0.1 g/l, and the activation of carboxyl end groups on the surface of GO sheets for covalent bond formation to immobilize hydrocarbon chains was performed using a mixture of 400 μM (EDC)/100 μM (NHS) at a volume ratio of 1:1. The BSA protein was immobilized onto the ends of GO sheets using EDC/NHS to activate and promote covalent bonding reactions between carboxyl groups on GO and –HN_2_ of BSA. The activated –COOH surface was then subjected to strong covalent immobilization via an amine (NH_2_)-coupling reaction with 20 μl of BSA protein at a concentration of 100 μg/ml. Finally, we used a centrifuge to repeatedly remove non-immobilized BSA protein on the GO surface. The GO-BSA antigen target procedure is shown in Fig. 1.

### Preparation of AuNPs and AuNP-GO Based on an Antibody Probe

**Fig. 2:**
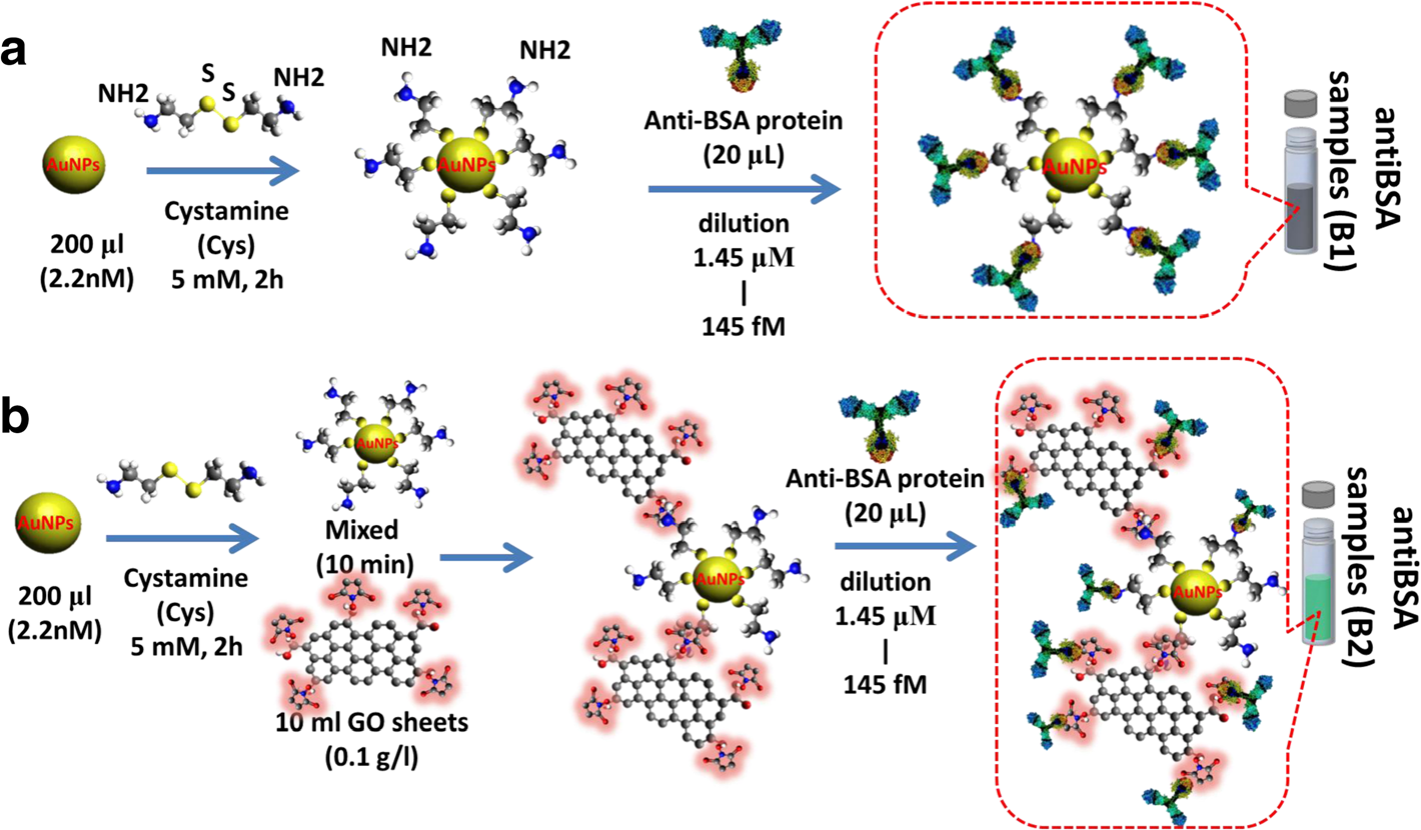
AuNP-antiBSA and AuNP-GO-antiBSA interactions. Preparation of the **a** AuNPs and **b** AuNP-GO based on antibody probe

We performed surface functionalization using Cys with 15-nm AuNPs in a volume of 200 μL. The AuNPs were chemically modified using a derivatized thiol self-assembled monolayer of Cys. The AuNPs were immersed in a solution of Cys (10 mM) for 2 h at room temperature. Because of the strong AuNP–S bond (thiol bond), Cys can easily connect to AuNPs and –NH_2_ groups exposed on the surface of AuNPs–Cys–NH_2_. We then added 20 μl of antibodies (antiBSA) of 100 μg/ml protein to immobilize the surface of the AuNPs, followed by repeated centrifugation to remove non-immobilized Cys on the surface of the AuNPs. Deionized water was then used to remove any non-immobilized AuNPs (Cys is an amino thiol structure that does not need to be activated by EDC/NHS). The Cys attached to the AuNPs had amine (–NH_2_) groups covalently coupled with carboxyl (–COOH) groups on the surface of antiBSA. Finally, we used repeated centrifugation to remove non-immobilized antiBSA protein from the surface of the AuNPs. A series of antiBSA protein concentrations were prepared by serial dilution from 100 μg/ml to 1 pg/ml. The preparation of the AuNP-antiBSA probe at different concentrations of antibodies is shown in Fig. 2a.

We used AuNPs prepared using the GO sheet method to modify the AuNP-GO nanocomposite. The AuNPs were prepared using the sodium citrate reduction method, and the size of the 60-nm AuNPs was modified using Cys (5 mM). We then used EDC/NHS to activate the –COOH groups on the surface of the GO sheets. The Cys attached to the AuNPs included –NH_2_ groups covalently coupled with –COOH groups on the surface of the GO sheets. The AuNPs on the surface of the GO sheets were immobilizing using a Cys linker to promote covalent bonding reactions between the AuNPs and GO. Covalent coupling offers a stable and easy method for bonding surface functionalization on GO sheets. The GO sheets were thoroughly rinsed with deionized water to remove unbonded GO at the AuNP-linker surface. The AuNP-Cys formed a new composite of AuNP-GO, followed by immobilization with different dilution concentrations from 100 μg/ml to 1 pg/ml of antibody (antiBSA) probe protein to form AuNP-GO-antiBSA, as shown in Fig. 2b.

### Characterization of AuNPs and GO Sheets

The dispersion and morphology of AuNP-GO sheets were characterized using a 300-kV field-emission gun transmission electron microscope (FEG-TEM; Tecnai G2F30S-Twin, Philips-FEI, Amsterdam, Netherlands) and a high-resolution transmission electron microscope (HR-TEM) on a FEI Tecnai G20 system (Hillsboro, OR, USA). The dispersion and morphology of the AuNP-GO and GO sheets were characterized using a JEOL JSM-7800F Prime Extreme-resolution Analytical Field Emission Scanning Electron Microscope (JEOL Inc., USA). The ultraviolet-visible (UV-vis) transmittance spectrum of a double beam spectrophotometer was observed using a UV-vis spectrophotometer (U-2900, Hitachi High-Technologies Corporation, Tokyo, Japan) with a wavelength from 200 to 1100 nm at room temperature. Raman measurements were performed using a microscopic Raman system (MRI, Protrustech Co., Ltd., Taiwan). An air-cooled spectrometer (AvaSpec-ULS2048L) with 1800 lines/mm grating and 50-μm slit was used as a detector. Fourier-transform infrared spectrometer (FTIR) measurements were made using a Bruker Vertex 80v spectrometer in attenuated total reflection (ATR) mode, and a DTGS detector (64 scans) with a resolution of 2 cm^− 1^ on a KBr pellet in a vacuum at a pressure of around 6 Pa. The Instrumentation Center at National Tsing Hua University provided support for this work. X-ray photoelectron spectroscopy (XPS) was performed using the facilities at the National Synchrotron Radiation Research Center, Hsinchu, Taiwan. The photoelectron spectroscopy experiments were performed using a 09A2 U5-spectroscopy beamline for XPS. Photons with fixed energies of 380 and 900 eV were used for C (1s) and Co (2p) throughout the core-level photoelectron spectroscopy experiments. The experiments were carried out in total-electron-yield mode at a 6-m high-energy spherical grating monochromator. The photons were incident to the normal surface, and photoelectrons were collected at an angle of 58° from the normal surface. The binding energies in all spectra referred to the Au 4f^7/2^ core level at 84.0 eV. After subtracting the linear background, the spectra were fitted with mixed Gaussian–Lorentzian functions based on a nonlinear least-square algorithm [65].

## Results and Discussion

### Structure and Morphology Analysis

**Fig. 3:**
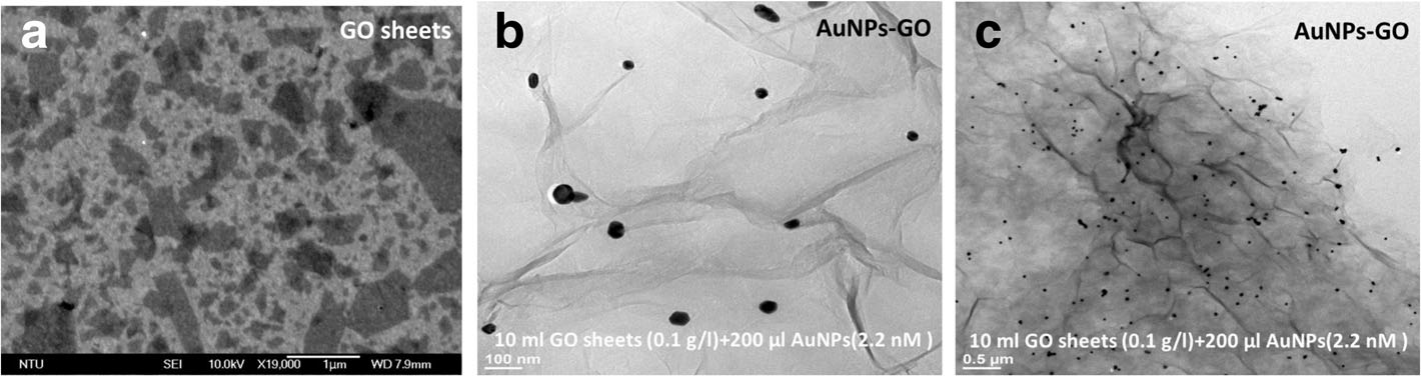
Surface morphology analysis of the nanocomposite. **a** SEM images of a GO sheet and **b** TEM image of AuNP-GO composite. **c** The TEM image of the ERGO AuNP-GO film

The size of the AuNPs depended on the nature of the reducing agent and conditions of formation and storage. The SEM analysis indicated that the GO sheets and AuNPs were uniformly attached on the GO surface, with an average AuNP size of 60 nm (Fig. 3). Figure 3a shows SEM images of the GO sheets on the Au film and that the GO sheets were less than 1 μm. Comparing GO and AuNP-GO, a gold element could be observed in the AuNP-GO composite. This indicated that the AuNPs had been successfully adsorbed on the wrinkled GO surface. The synthesized AuNP-GO hybrids were characterized using TEM as shown in Fig. 3b. Figure 3b, c shows the synthesis conditions. The concentration of 10 ml GO sheet solution was 0.1 g/l, and 200 μl of AuNP solution had a concentration of 2.2 nM. The TEM images in Fig. 3a with a 100-nm scale bar and Fig. 3b with a 0.5-μm scale bar show different amplifications of the size and clearly show that the AuNPs were immobilized on the surface of the GO sheets. The AuNPs had a spherical shape, suggesting that carboxylic functionality on the surface of the GO sheets may play an important role in the formation of chemical covalent bonds with the AuNPs as shown in Fig. 2b.

### Characterizations of GO by XPS, Raman, and FTIR Spectroscopy

**Fig. 4:**
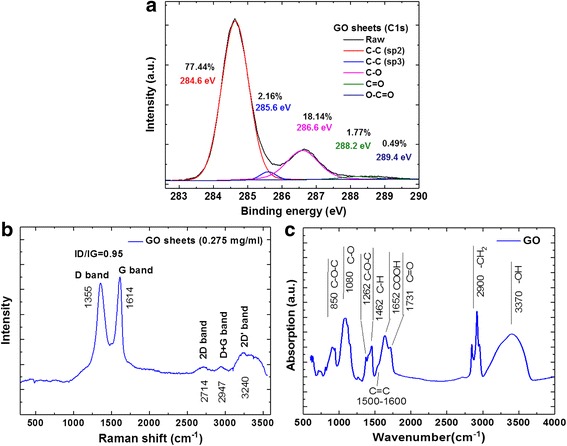
Spectral analysis of GO sheets. **a** XPS high-resolution scan in the C1s region, **b** Raman, and **c** FTIR

Figure 4a shows the high-resolution C 1s XPS spectral analysis of the GO sheets. The C1s of the highest intensity at a binding energy of 284.6 eV corresponded to carbonyl functional groups for C–C (sp2), and the peaks at 285.6, 286.6, 288.2, and 289.4 eV corresponded to C–C (sp3) in aromatic rings, C–O in hydroxyl and epoxy groups, C=O in carbonyl groups, and O–C=O in carboxyl groups, respectively. The C 1s XPS spectra of the GO sheets on a gold film substrate showed that the relative atomic percentages of C–C (sp2), C–C (sp3), C–O, C=O, and O–C=O were 77.44, 2.16, 18.14, 1.77, and 0.49%, respectively [66]. Figure 4b shows Raman spectral analysis of the GO sheets in NaCl solution with spectral feature peaks at 1614 cm^− 1^ (G band), 1355 cm^− 1^ (D band), 2714 cm^− 1^ (2D band), 2947 cm^− 1^ (G + D band), and ~ 3240 cm^− 1^ (2D’ band) [38, 67]. The formation of the GO sheets was further analyzed to elucidate the properties of various oxygenated species using ATR-FTIR spectra as shown in Fig. 4c. This ATR-FTIR spectra also revealed several characteristic peaks of GO; C–O at 850 cm^− 1^ due to (C–O–C) epoxide vibrations, C–O at 1080 cm^− 1^ due to (C–O) alkoxy stretching vibration, C=C at 1500~1600 cm^− 1^ due to aromatic C=C bonds, and C–O at 1260 cm^− 1^ due to (C–O–C) epoxide asymmetric vibrations. The carboxylic groups at the edges of the GO sheets showed –COOH stretching vibrations, with peaks at 1652 and 1731 cm^− 1^ corresponding to C=O stretching vibrations from carbonyl groups. The spectra also showed three peaks at 2900 cm^− 1^ related to asymmetric and symmetric stretching vibrations of –CH_2_ and a deformation peak at 1462 cm^− 1^ due to alkene groups (C–H) located on the plane of GO. FTIR spectra of GO showed a large range of absorption broad band peaks of C–OH and H_2_O vibrations at 3370 cm^− 1^ due to stretching vibrations [67].

### Analysis of GO and AuNP with Protein Interaction Properties

**Fig. 5:**
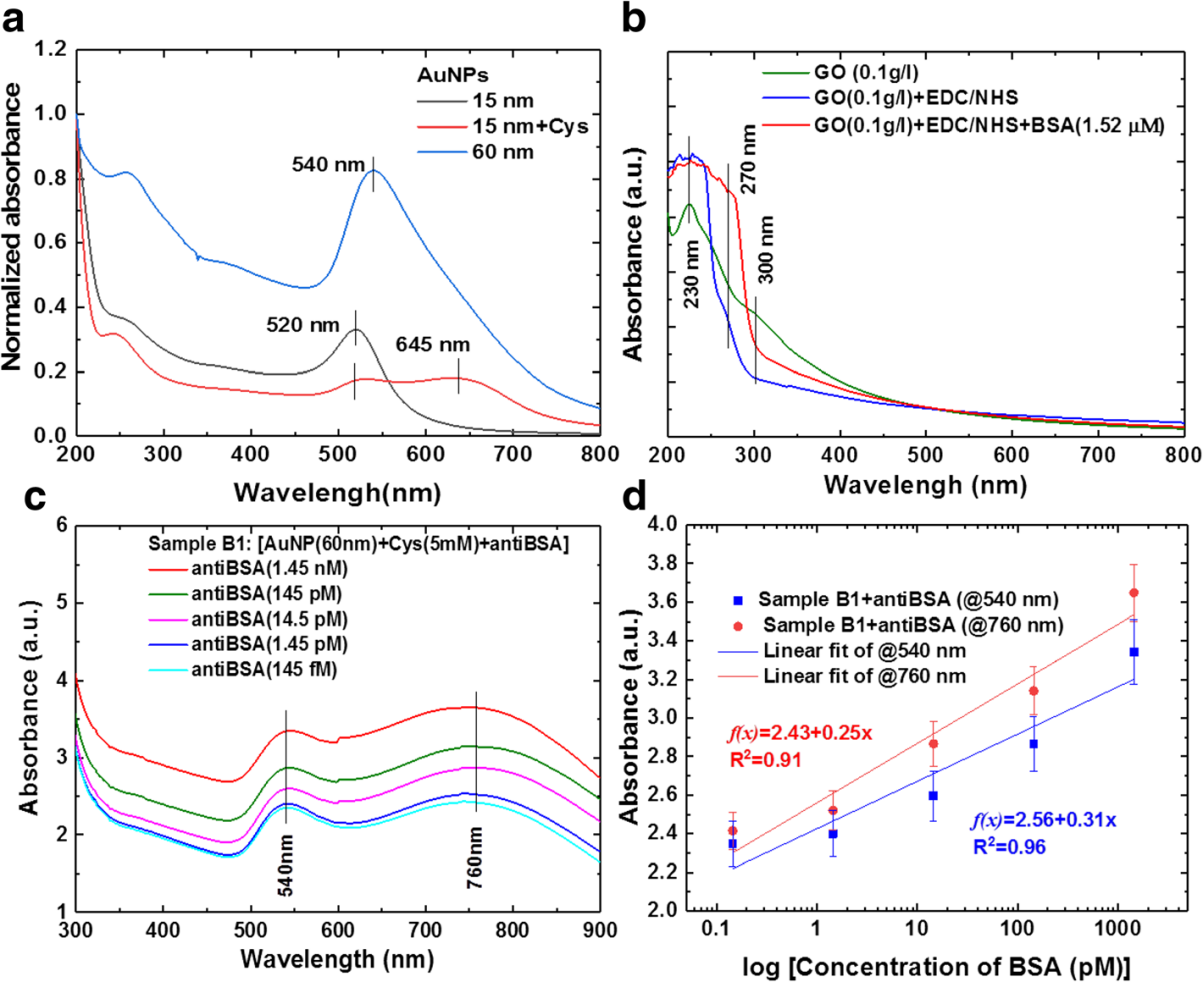
Analysis of UV-vis absorption spectra for AuNP with antiBSA interaction response. **a** SP absorption spectra of AuNPs, **b** GO-bound BSA, **c** AuNP-anti-BSA probe, and **d** Calibration curves for AuNP with antiBSA interaction response at dilution different concentrations of antiBSA protein from 1.45 nM~145 fM

UV-vis spectra of aqueous GO and AuNPs with protein interaction dispersions are presented in Fig. 5. The AuNPs had high extinction coefficients and a unique size depending on the surface plasmon (SP) absorption bands. The modified AuNPs of two different dimensions were 15 and 60 nm for 520 and 540 nm of SP absorption spectra [68, 69]. Figure 5a shows the AuNP (520 nm) solution modified with Cys at a concentration of 5 mM with the AuNP-Cys extinction band [69]. The UV-vis spectra of aqueous GO dispersions yielded two absorption peaks: the maximum at 230 nm corresponding to the π–π* plasmon peak of aromatic C–C bonds in differently sized aromatic sp2 clusters, and the shoulder at 300 nm corresponding to the n–π* plasmon peak owing to the presence of epoxide and carbonyl (C=O) bonds (Fig. 5b) [9, 20]. The UV-vis spectra of GO–EDC/NHS and GO–EDC/NHS–BSA solution (Fig. 5b) demonstrated that GO–EDC/NHS and GO–EDC/NHS–BSA showed a peak at about 270 nm, which was probably due to strong interactions between GO and amine groups [70, 71]. Figure 5c shows the absorption spectra of the bonds for different concentrations of antiBSA with AuNPs (60 nm). Figure 1b shows the synthesis solution of the AuNP-antiBSA probe (sample B1). The wavelengths had obvious absorption peaks at 540 and 755 nm, with the wavelength at 540 nm being mainly caused by the AuNPs (60 nm), and the peak at 755 nm corresponding to a AuNP+Cys+antiBSA combination absorption peak. This result showed that an increase in antiBSA concentration induced a gradual increase in the absorbance at 540 nm and a continuous increase in the near-IR absorption at 755 nm. The different antiBSA-binding interactions at the surface of the AuNPs caused changes in the surface refractive index, which in turn was transduced into absorbance intensity of the LSPR. The LSPR band was affected by particle size. The AuNPs showed strong SPR bands in the visible region. With increases in the refractive index of the medium-sized particles, the shift in SPR peak positions could be tuned from the visible to the near-infrared. In the protein interaction experimental results, we mixed AuNP-antiBSA as shown in Fig. 5d. Sensitivity was determined by plotting the LSPR wavelength of the 500–760 nm band as a function of the measured refractive index. We then used dilution different concentrations of antiBSA protein from 1.45 nM to 145 fM and mixed them in a 200-μl volume. The changes in 540 and 760 nm absorption were caused by the different concentrations of antiBSA and AuNP. Spectral measurements were then made 5 min later, which showed different concentrations of light intensity absorption, and an absorption peak of 60 nm for the AuNPs was observed at 540 and 755 nm. These results were consistent with the AuNP-antiBSA absorption calibration curves. The calibration curves were fitted by *y* = 2.43 + 0.25× (correlation coefficient, *R*^2^ = 0.91) for the 540-nm absorption peak, and *y* = 2.56 + 0.31× (correlation coefficient, *R*^2^ = 0.96) for the 760-nm absorption peak, where *x* is the concentration of antiBSA and *y* is the optical absorbance.

### Analysis of AuNP-antiBSA and AuNP-GO-antiBSA Based on Immunoassay Interactions

**Fig. 6:**
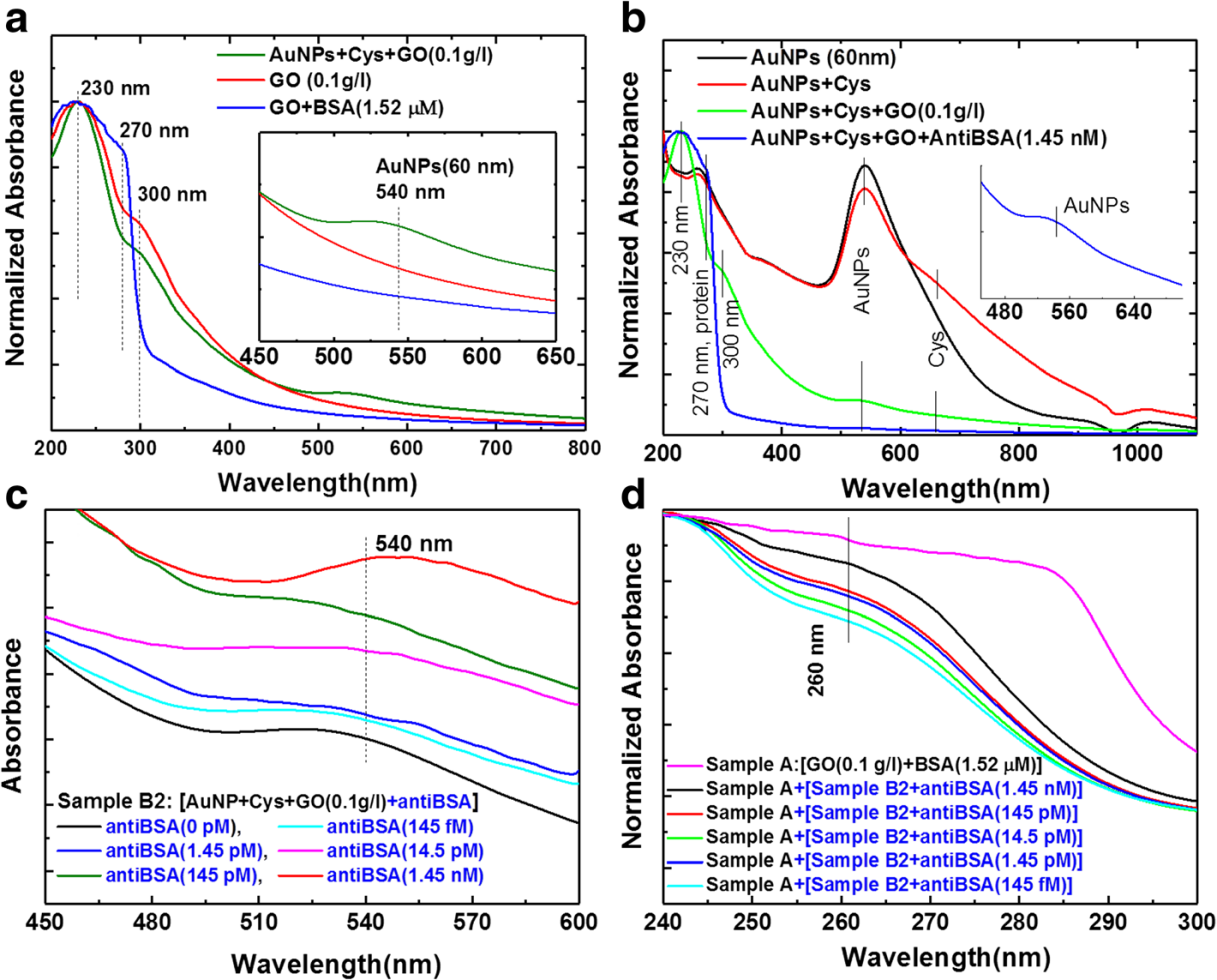
Analysis of UV-vis absorption spectra for immune response. **a** AuNP-bound GO and GO-bound BSA, **b** AuNPs and GO-bound anti-BSA, **c** AuNP-GO-antiBSA probe, and **d** AuNP-GO-antiBSA probe and GO-BSA target for immune response

In order to understand the immunological detection mechanism of GO and AuNP-GO nanocomposites, spectral analysis for binding reactions was performed as shown in Fig. 6. Figure 6a shows the UV-vis absorption spectra of the AuNP-GO and GO-BSA nanocomposites. For the GO sheet (0.1 g/l) solution, there was a peak at about 230 nm [70] and a shoulder at around 300 nm, and the GO–BSA conjugates showed an absorption peak at about 270 nm and a peak at about 230 nm [9, 20, 70]*.* In the combination of AuNP-GO nanocomposites, three absorption peaks were noted at 230, 300, and 540 nm, respectively. The π–π stacking or covalent bonding interactions between AuNPs and the GO sheet surface were the main driving force anchoring the AuNPs onto the highly biocompatible GO materials. The GO sheets were made to congregate in the AuNPs, resulting in a strong absorption band of 200–300 nm. Therefore, the absorption of the GO sheets was much greater than the absorption of AuNPs in the visible light band. Figure 6b shows that the UV-vis spectra of the AuNP absorption peak were at 540 nm [50, 68, 69]. The absorption peaks were at 540 and 660 nm for AuNP+Cys conjugates; 230, 300, 540, and 660 nm for AuNP+Cys+GO conjugates; and 230, 270, 540 and 660 nm for AuNP+Cys+GO+antiBSA conjugates. The GO sheets had two absorption peaks at 230 nm (π–π* plasmon peak) and 300 nm (n–π* plasmon peak). A shift in the absorption wavelength was noted, and this absorbance shift was considered to indicate confirmation of antiBSA (0 fM ~ 1.45 nM) absorption onto the AuNP+Cys+GO surface. Figure 6c shows the synthesis of the solution of the AuNP+Cys+GO+antiBSA probe (sample B2) as in Fig. 2b. The increase in antiBSA concentration was relatively high at 540 nm. Figure 6c shows different concentrations of light intensity absorption, and an absorption peak of 60 nm for the AuNPs was observed at 540 nm. The increase in antiBSA concentration was relatively high at 540 nm. This result showed that AuNP-GO could enhance the plasmon absorption characteristics at 540 nm when the antiBSA concentration was increased. In addition, in the immunoassay experiment, we mixed the GO-BSA (1.52 μM) target (sample A) and AuNP+Cys+GO+antiBSA probe as shown in Fig. 6d. In addition to hydrophobic and π–π interaction characteristics of the GO sheets, covalent bonds between proteins and carboxyl groups on the GO sheets also supported surface adhesion. This result was likely due to the AuNP+GO-antiBSA hybrid structure to form a stable immune response with other proteins on GO-BSA. The wavelengths had an obvious absorption peak at 260 nm. In addition to hydrophobic and π–π (π–π* plasmon peak) interaction characteristics of the GO sheets, covalent bonds between proteins and carboxyl groups on the GO sheets also supported surface adhesion. Before and after BSA and antiBSA bonding, the π–π* plasmon peak values of GO (230 and 270 nm) were significantly shifted, which proved that BSA and antiBSA were positively bonded.

**Fig. 7:**
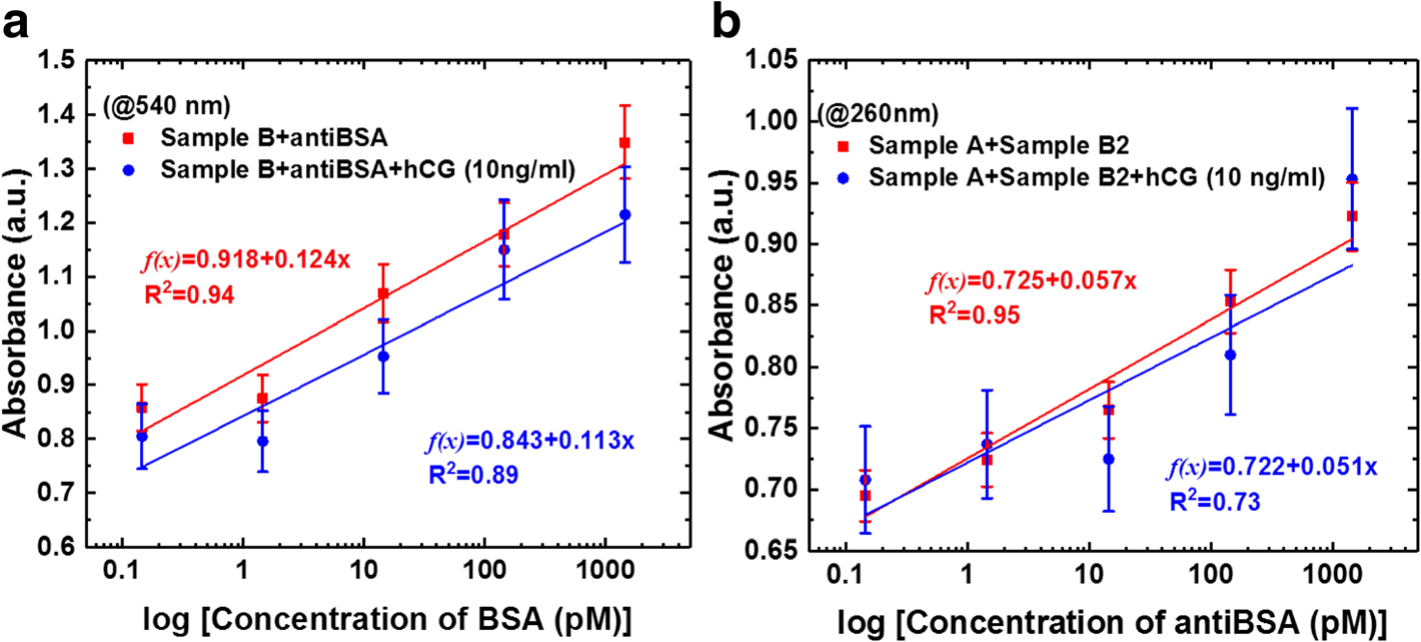
Comparison of the calibration curves of sensing responses obtained at different protein concentrations. **a** Calibration curves for AuNP-GO probe with antiBSA interactions. **b** Calibration curves for GO and GO-AuNPs based on an immunoassay

Figure 7 shows that these results were in good agreement with the calibration curves. Detailed analysis of the above experimental results (Fig. 6c, d) showed that the sensing responses to the corresponding average inaccuracies of absorbance were 1.3487, 1.1776, 1.0698, 0.8755, and 0.8588 (Fig. 7a) and 0.9226, 0.8535, 0.7649, 0.7243, and 0.695 (Fig. 7b), corresponding to 1.45 nM, 145 pM, 14.5 pM, 1.45 pM, and 145 fM protein concentrations, respectively. Figure 7a shows that the linear regression of the calibration curves was *f(x)* = 0.918 + 0.124*×* (correlation coefficient, *R*^2^ = 0.94) for the AuNP-GO probe with antiBSA interactions, where *x* is the protein concentration and *y* is the optical absorbance. In addition, Fig. 7b shows that the linear regression equation of the fitted curve was *f(x)* = 0.791 + 0.057*×* (correlation coefficient, *R*^2^ = 0.954) for GO and AuNP-GO based on the immunoassay.

During the quantitation experiment, we added a fixed concentration of 10 ng/ml of human chorionic gonadotropin (hCG) protein to act as an interferer. The results showed that the fixed interferer hCG protein on the immunoassay calibration curves were fitted by *f(x)* = 0.843 + 0.113× (correlation coefficient, *R*^2^ = 0.89) for the AuNP-GO probe with antiBSA interactions (Fig. 7a), and *f(x)* = 0.722 + 0.051*×* (correlation coefficient, *R*^2^ = 0.73) for the GO and AuNP-GO based on the immunoassay (Fig. 7b).

Furthermore, our experimental results showed that the detection strategy allowed for surface regeneration with no loss in specificity (four regenerations) and that it could also be used to detect antiBSA protein with dynamic responses ranging from 1.45 nM, 145 pM, 14.5 pM, 1.45 pM, 145 fM, and 0 fM. The results demonstrated that with a decreased concentration of antiBSA (from 1.45 nM to 145 fM) and even without the presence of antiBSA (0 fM), the spectral absorption intensity did not change the minimum level of quantitation. The hCG protein interfered with the antibody recognition in the immunoassay to a limited extent, possibly due to non-specific adsorption. This implies a very low cross-reactivity of the hCG protein and non-specific interactions at a low adsorption. In the practical quantitative analysis with immunoassays, a LOD of 145 fM for antiBSA was achieved in both buffer and interference protein samples.

## Conclusions

We successfully demonstrated a GO-bound AuNP biocompatible nanocomposite in a biosensing mechanism in a rapid and label-free immunoassay for biomolecule interactions. The results showed that the AuNP-GO nanocomposite was biocompatible and exhibited LSPR extinction to biomolecules, which could promote the absorption spectra characteristic peaks, accelerate the reaction of molecules, and enhance the stability of chemical covalent bonds during immobilization. For the detection of antiBSA protein, the limit of detection of the GO and AuNP-GO based on the immunoassay was as low as 145 fM. Among the AuNP-GO biosensors, GO immobilized in the AuNP-GO nanocomposite showed the highest bioaffinity, with good sensitivity, low detection limit, and fast response toward the protein immunoassay. The results of our experiments showed that a fixed concentration 10 ng/ml of hCG protein as an interferer did not affect the test response. Given the growing trend of applying biosensors in POCT, LSPR for AuNP-GO nanocomposite technology is a highly promising and versatile tool for use in immunoassays. Combining the properties of AuNPs and GO sheets to develop new nanocomposites for the synthesis of smart materials shows promise for the development of user-friendly diagnosis applications. In the future, AuNP-GO nanocomposites may be used in innovative immunoassays, rapid detection reagents, and miniaturization, which may in turn make LSPR technology an irreplaceable tool for routine clinical analysis and POCT diagnostics.

## Additional file

Additional file 1: **Figure S1**. Comparison of the three different temperatures for the synthesis of AuNPs as shown by the absorption spectrum. (DOCX 43 kb)

## Abbreviations

Ag: Silver
AntiBSA: Bovine serum albumin antibody
Au: Gold
AuNP: Gold nanoparticle
BSA: Bovine serum albumin
Cys: Cystamine
EDC: 1-Ethyl-3-(3-dimethylaminopropyl)carbodiimide
FEG-TEM: Field-emission gun transmission electron microscope
FTIR: Fourier-transform infrared spectrometer
GO: Graphene oxide sheet
HR-TEM: High-resolution transmission electron microscope
LOD: Limit of detection
LSPR: Localized surface plasmon resonance
MOA: 8-Mercaptooctanoic acid
NHS: *N*-Hydroxysuccinimide
Pd: Palladium
POCT: Point-of-care testing
Pt: Platinum
RGO: Reduction graphene oxide
SAMs: Self-assembled monolayers
SERS: Surface-enhanced Raman scattering
UV-vis: Ultraviolet-visible
XPS: X-ray photoelectron spectroscopy
ZnO: Zinc oxide
