# Supplementary material for: Enhanced Plasmonic Biosensors of Hybrid Gold Nanoparticle-Graphene Oxide-Based Label-Free Immunoassay
Source: Nanoscale Res Lett. 2018 May 16;13:152. doi: 10.1186/s11671-018-2565-7 (PMC5955872; doi:10.1186/s11671-018-2565-7)
Supplement: Supplementary file 1 — Figure S1. Comparison of the three different temperatures for the synthesis of AuNPs as shown by the absorption spectrum. (DOCX 43 kb) [file 11671_2018_2565_MOESM1_ESM.docx]

**Supplementary material**

**Enhanced plasmonic biosensors of** **hybrid gold nanoparticle-graphene oxide-based label-free immunoassay**

Nan-Fu Chiu*, Chi-Chu Chen, Cheng-Du Yang, Yu-Sheng Kao, Wei-Ren Wu

Corresponding author:

Address：Laboratory of Nano-photonics and Biosensors, Institute of Electro-Optical Science and Technology, National Taiwan Normal University, No. 88, Sec. 4, Ting-Chou Road, Taipei 11677, Taiwan

E-Mail：[nfchiu@ntnu.edu.tw](mailto:nfchiu@ntnu.edu.tw)

TEL: +886-2-77346722 FAX: 886-2-8663-1954

- We have described remanufacturing AuNPs under different temperature conditions as recommended by the reviewer. We designed three groups of different temperature conditions (550, 400 and 100 °C). The detailed conditions were as follows:

1. A volume of 15 mL of HAuCl_4_·3H_2_O solution containing 1 mM of Au was refluxed, and 1.8 mL of 38.8 mM sodium citrate (Na_3_C_6_H_5_O_7_) solution was added to the boiling (550 °C, 1100 rpm) solution. Reduction of the Au ions by the citrate ions was complete after 5 minutes, and the solution was boiled for another 30 minutes (550°C, 900 rpm) and then left to cool to room temperature.
2. A volume of 15 mL of HAuCl_4_·3H_2_O solution containing 1 mM of Au was refluxed, and 1.8 mL of 38.8 mM sodium citrate (Na_3_C_6_H_5_O_7_) solution was added to the boiling (400 °C, 1100 rpm) solution. Reduction of the Au ions by the citrate ions was complete after 5 minutes, and the solution was boiled for another 30 minutes (400 °C, 900 rpm) and then left to cool to room temperature.
3. The same concentration and volume was added to the boiling (100 °C, 1100 rpm) solution. Reduction of the Au ions by the citrate ions was complete after 120 minutes, and the solution was boiled for another 30 minutes (100 °C, 900 rpm) and then left to cool to room temperature.

The experimental results (Figure S1) of these three different temperatures for the reduction of AuNPs showed that the absorption spectrum was consistent at a wavelength of 520 nm. However, the most important condition for the reduction of the Au ions was that the mixture of HAuCl_4_·3H_2_O and sodium citrate solution had to be boiling.


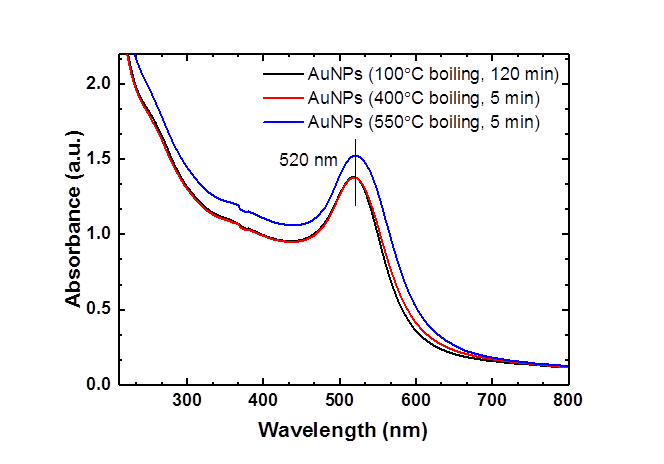


Figure S1. Comparison of the three different temperatures for the synthesis of AuNPs as shown by the absorption spectrum.
